# Vascular diaphragmatic hernia in a patient with cirrhosis first case report

**DOI:** 10.1186/2047-783X-17-16

**Published:** 2012-06-14

**Authors:** Mihai I Lazăr, Daniela Adriana IF Ion

**Affiliations:** 1 National Institute for Infectious Diseases Prof. Dr. Matei Bals, Str. Calistrat Grozovici, No. 1, 2nd District, Bucharest, Romania; 2 University of Medicine and Pharmacy Carol Davila, Str. Calistrat Grozovici, No. 1, 2nd District, Bucharest, Romania

**Keywords:** Cirrhosis, Diaphragmatic hernia, Portal hypertension, Vascular hernia

## Abstract

We report the case of an adult patient recently diagnosed with cirrhosis. The ultrasound evaluation described a multinodular inhomogeneous liver, requiring a magnetic resonance imaging scan for further characterization. The performed magnetic resonance imaging examination confirmed the diagnosis of cirrhosis associated with portal hypertension and detected a vascular left transdiaphragmatic hernia. Although various types of diaphragmatic hernias have been described - congenital or acquired - to the best of our knowledge, this type of pathology has never been reported.

## Background

Diaphragmatic hernias appear secondary to structure or insertion abnormalities of the diaphragm and can be divided in two major categories - congenital and acquired hernias. They represent a permanent or temporary displacement of abdominal structures (stomach, spleen, liver, small or large bowel) into the thoracic cavity or a transdiaphragmatic extension of pathological entities, such as a pancreatic pseudocyst and mediastinal or retroperitoneal lipomas (if associated with dehiscence of the diaphragmatic crus or other insertion abnormalities).

Congenital hernias have a prevalence in the newborn population of one in 3,000 [1] and can be categorized into three types: posterolateral hernias (Bochdalek); retrosternal hernias (Morgagni-Larrey); and hiatal hernias (embryological mechanisms: septum transversum defects; failed development of the pleuroperitoneal folds; and improper migration of the diaphragmatic musculature) [2]. Acquired diaphragmatic hernias have iatrogenic or post-traumatic etiology, after a significant increase of pressure in the abdominal cavity, such as in the case of a penetrating or blunt trauma (the most common cause) [3]. Situations inducing a moderately increased pressure, such as severe and persistent cough or large ascites, are not usually related to diaphragmatic hernias; however, supplementary pressure can be exerted on abdominal weak points, explaining the herniation of abdominal structures.

In our patient, the herniated structure was an anastomotic vessel connecting the left branch of his portal vein and the venous circulation of the left abdominal wall.

## Case presentation

We present the case of a 45-year-old white man, with a history of alcohol use (100 to 200 mL alcohol/day for more than 20 years), who was recently diagnosed with cirrhosis after an ultrasound examination. An echographic examination was performed prior to his admission to our clinic, which revealed a pseudonodular, inhomogeneous liver with fibrotic changes, an enlarged main portal vein with decreased velocity (7.3 cm/s) and multiple venous collateral circulations trajects (recanalized umbilical vein, enlarged mesenteric and perisplenic veins). These findings were suggestive of cirrhosis with vascular decompensation. The spleen evaluation showed a normal size (11 cm) and multiple microcalcifications, interpreted as remnants of prior inflammatory lesions. No other pathological findings were reported.

In order to exclude the presence of neoplastic lesions in the liver, additional investigations were required and the patient was referred to our clinic for further evaluation.

During the clinical examination, the patient was conscious, cooperative, presenting a slightly decreased alveolar murmur in the pulmonary bases without abnormal breath sounds, abdominal discomfort on palpation in the right hypochondrium and a slightly enlarged liver, palpable in the epigastrium. Superficial lymph nodes were non-detectable. Collateral venous circulation was visible on both flanks.

A thorough blood work-up revealed the following: normal transaminases levels (aspartate transaminase, 51 IU/L, alanine transaminase, 70 IU/L); a slight increase in gamma-glutamyltranspeptidase (117 U/L) with alkaline phosphatase in the normal range (73 U/L); mild thrombocytopenia (130,000/mm^3^); and normal range values for red blood cells (5.28 × 10^6^/mm^3^), hemoglobin (13.8 g/dL), hematocrit (49.6 %), mean corpuscular volume (93.9 fL), mean corpuscular hemoglobin (31.8 pg) and red blood cell distribution width (12.5 %), unconjugated bilirubin (0.5 mg/dL), conjugated bilirubin (0.6 mg/dL), plasma total proteins (8.1 g/dL) and albumin (4.65 g/dL). He scored F3 in a FibroTest evaluation, with no inflammatory syndrome (fibrinogen, 463 mg/dL; neutrophilic leukocytes, 5,400/mm^3^, K^+^ and Na^+^ were in the normal range. Immunologic tests for viral hepatitis were negative (hepatitis B surface antigen – AgHBs I negative; anti-HCV antibody - AcHVC I negative) and coagulation tests showed no alteration (international normalized ratio, 0.96; activated partial thromboplastin time 33 seconds).

A magnetic resonance imaging (MRI) scan was performed in order to assess the hepatic nodules described on the ultrasound examination, to exclude a possible hepatocarcinoma and to detect other possible associated pathologies.

The MRI sequences showed the inhomogeneous structure of the liver, with a pseudonodular aspect, without signal abnormalities suggestive for neoplastic pathology as well as an irregular liver contour and increased dimensions of the left hepatic lobe and caudate segment. His spleen was normal size but with an inhomogeneous structure presenting small granular low signal images on both T1- and T2-weighted sequences, compatible with the microcalcifications described on ultrasound. Multiple collateral vascular trajects with perisplenic, periumbilical and perigastric topography were also observed, representing portosystemic collateral circulation.

**Figure 1:**
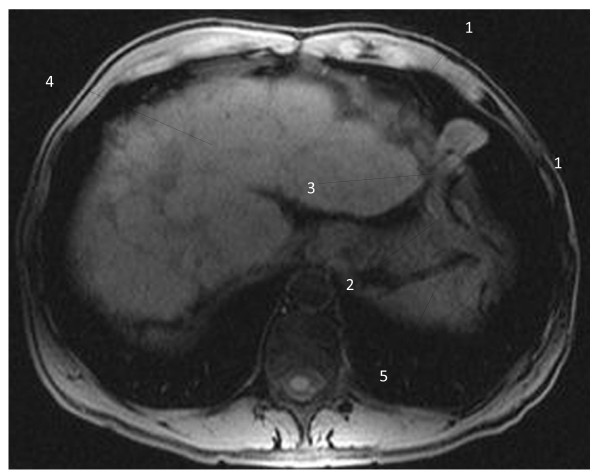
**T1-weighted magnetic resonance image of the vascular diaphragmatic hernia (non-contrast image), axial view.** 1 - Left diaphragm muscle; 2 - descending part of the collateral aberrant vein; 3 - diaphragmatic defect corresponding to the base of the vascular loop; 4 - liver; 5 - spleen.

**Figure 2:**
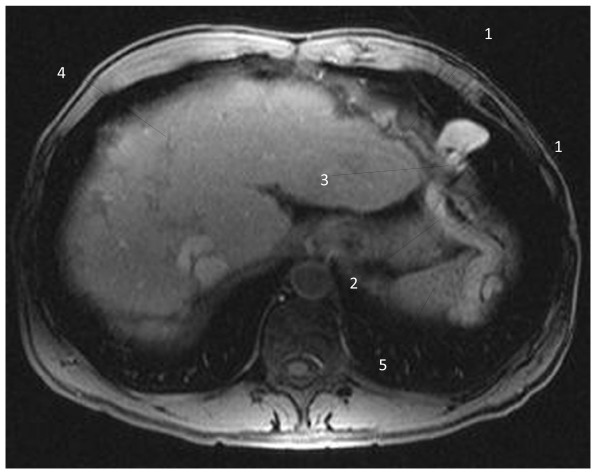
**T1-weighted magnetic resonance image of the vascular diaphragmatic hernia (after contrast administration), axial view.** 1 - Left diaphragm muscle; 2 - descending part of the collateral aberrant vein; 3 - diaphragmatic defect corresponding to the base of the vascular loop; 4 - liver; 5 - spleen.

**Figure 3:**
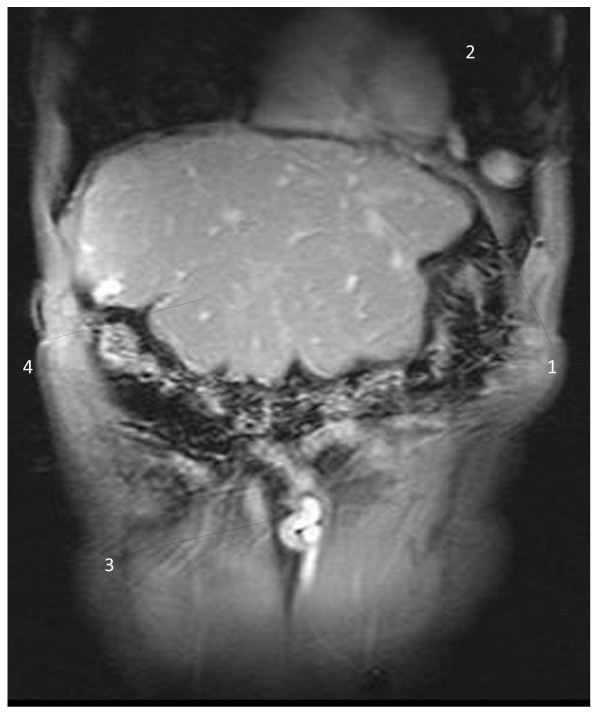
**T1-weighted magnetic resonance image of the vascular diaphragmatic hernia (after contrast administration), coronal view.** 1 - Left diaphragm muscle; 2 - thoracic part of the herniated vein; 3 - recanalized umbilical vein; 4 – liver.

**Figure 4:**
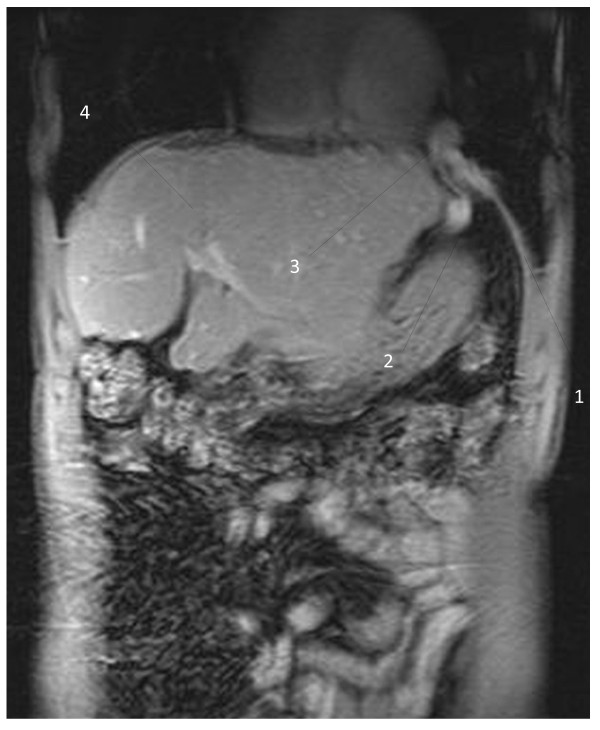
**T1-weighted magnetic resonance image of the vascular diaphragmatic hernia (after contrast administration), coronal view**. 1 - Left diaphragm muscle; 2 - abdominal part of the herniated vein; 3 - diaphragmatic defect corresponding to the base of the vascular loop; 4 - liver.

The interesting finding was an aberrant vein with its origin in the left hepatic portal branch, which follows a horizontal trajectory to the left, compressed between the enlarged left hepatic lobe and the inferior side of the left diaphragm; the vein herniated into the left thoracic cavity, described a loop and returned into the abdominal cavity (Figure 1); it passed between the spleen and the diaphragm muscle, and led inferolaterally into the left abdominal wall. T1-weighted images with contrast enhancement detected no signal abnormality in the liver parenchyma and confirmed the presence of the aberrant vascular trajectory (Figure 2); no thrombotic images were detected in the portal or collateral circulation. The coronal reconstructions (Figures 3 and 4) allow a better view of both abdominal and thoracic segments of the herniated vein, of its extension into the thoracic cavity and also of the recanalized umbilical vein (Figure 3). Patient received propranolol (20 mg/day) for the portal hypertension, lactulose (30 mL/day) to prevent portal encephalopathy, omeprazol (20 mg/day) in the morning to prevent bleeding from the gastric and esophageal varices, and hepatoprotectors (silibinum; 500 mg/day). He was further addressed to a thoracic surgery department for a consultation and treatment; he refused the intervention, remaining under periodic surveillance.

## Discussion

The negative immunological tests corroborated with the imaging investigations (pseudonodular aspect of the liver, with irregular liver contour, increased dimensions of the left hepatic lobe and caudate segment, portosystemic collateral circulation) and the elevated level of gamma-glutamyltranspeptidase suggest liver cirrhosis with a possible alcohol use etiology.

The manifestations of acquired diaphragmatic hernias are correlated with their size and content; they include circulatory and respiratory depression secondary to decreased function of the diaphragm and to compression of the lungs by the herniated abdominal structures. In the case of small diaphragmatic hernias, the symptoms are absent or mild (nonspecific gastrointestinal complaints), often ignored by the patients; the diagnosis is met when the size of the hernia increases and the symptomatology becomes more important or complications such as strangulation or dyspnea occurs.

Hernias represent a weakness in the abdominal wall that can evolve into a localized defect, allowing abdominal structures covered with peritoneum to protrude: the protruding abdominal structure (a collateral vein) can be observed in Figures 1, 2, 3 and 4. The particularity and originality of this case report is represented by an unusual pathology, a vascular transdiaphragmatic hernia, described, to the best of our knowledge, for the first time. A possible explanation for this pathology can be found in the process of diaphragm formation. During the embryologic period, weak points in the structure of the diaphragm muscle may appear (related to migration or fusion abnormalities), which are too small for the large abdominal organs to herniate and therefore they remain undetected, but large enough to allow the herniation of small structures. If there is any increased pressure in the abdominal cavity (caused by chronic cough or large ascites in patients with cirrhosis), the anatomic structures lying in the proximity of a weak point may protrude through the weak point into the thorax. In our case, the large anastomotic vein was compressed between two rigid planes - the superior side of the hypertrophied left liver lobe (presenting decreased elasticity due to the fibrotic changes) and the inferior side of the diaphragm.

In order to establish a positive diagnosis of diaphragmatic hernia, two main elements should be present: a diaphragmatic defect and an abdominal structure that passes through the defect into the thorax. When analyzing the vessel on the contrast enhanced images (Figure 2), we can observe a continuous contour, similar wall thickness of the vessel in both segments, thorax and abdomen, and a MR-signal compatible with lung parenchyma surrounding the thoracic part of the vessel, thereby excluding a diaphragmatic relaxation. The vessel is situated posterior and inferior in rapport with the diaphragm, passes through the diaphragmatic defect into the thoracic cavity and then returns into the abdomen. To obtain an optimal evaluation of the diaphragmatic defect, either a laparoscopy/laparotomy or radio-imaging examinations (computed tomography (CT) or MRI) could be performed. In our case, the patient refused the surgical intervention; the diaphragmatic defect can be observed in Figures 1, 2 and 4 - corresponding to the base of the vascular loop.

The relevance of this particular case is related to potential diagnostic problems, therapeutic implications and possible complications that may occur in the natural evolution of the disease, such as strangulation, thrombosis or rupture with secondary hemothorax, which are difficult to treat and diagnose if this diagnosis has not previously been considered.

Pleural effusions are very common in patients with cirrhosis, especially in the late stages, and usually they have an insidious, asymptomatic evolution, correlated with the protein serum level. If the liquid quantity increases in a short time, another etiology (usually infectious) must be considered. In this pathological context, if specific pulmonary infectious findings are absent and the patient presents hypovolemic symptomatology, a rupture of the herniated collateral vein must be also considered. Another implication of this finding is related to common therapeutic practices - if a pleural effusion is to be evacuated by puncture in a patient with cirrhosis, the existence of a vascular hernia represents a contraindication due to the possible interception of the aberrant vessel and secondary hemothorax.

The presence of a vascular diaphragmatic hernia also extends the differential diagnosis when analyzing a thoracic radiography, in cases of pulmonary opacities situated in the base of the lungs.

D’Amico and Luca [4] have shown that esophageal and/or gastric varices eventually develop in all cirrhotic patients and that, once developed, they tend to increase in size and to bleed. The management of portal hypertension is aimed first of all at the prevention of variceal bleeding and treatment of acute bleeding when it occurs - in this instance, endoscopic sclerotherapy (EST) and band ligation of esophageal varices (EVL) are widely considered the treatment of choice. However, after the surgical treatment of varices, changes in the portal blood flow occur that lead to the increased size of the remaining collateral veins. Sarin *et al*. [5] communicate an aggravation of portal hypertensive gastropathy after EVL and EST due to a sudden increase of the blood flow in the venous collaterals. Based on these elements, if a vascular hernia is present (as in our case), an adjustment of the surgical attitude is necessary when the patient undergoes EST or EVL, to prevent an eventual rupture of the aberrant vein.

Usually the collateral vessels treated in cases of patients with cirrhosis are the gastro-esophageal varices; our case report adds another collateral vessel to the surgical indication list, in order to avoid possible complications.

The standard evaluation for varices is represented by esogastro-duodenoscopy (EGD), which should be carried out for every patient at the time when cirrhosis is diagnosed. Garcia-Tsao *et al*. [6] recommend, for patients with cirrhosis and no varices, initial EGD should be repeated in 3 years or annually if there is evidence of hepatic decompensation; for patients with cirrhosis and small varices that have not bled, it should be repeated in 2 years or annually if there is evidence of hepatic decompensation; and in patients with cirrhosis and medium or large varices that have not bled, EGD should be performed 1 to 3 months after obliteration and then every 6 to 12 months to check for variceal recurrence. However, in order to evaluate the entire collateral circulation in a patient with portal hypertension, other investigative methods are also required – ultrasound, CT or MRI. The ultrasound evaluation in our case described multiple perisplenic venous trajects, but could not detect the abnormal vessel herniated in the left thoracic cavity; it is important in such cases, for an accurate assessment of the patient, to corroborate the ultrasound data with additional imaging investigations (MRI or CT), which can also provide further information regarding the hepatic structure and/or other associated pathologies.

## Conclusion

Vascular transdiaphragmatic hernia, although a very rare pathological entity, may complicate the evolution of a cirrhotic patient. Such a possibility must be considered for a proper management of the disease.

## Consent

Written informed consent was obtained from the patient for publication of this case report and any accompanying images. A copy of the written consent is available for review by the Editor-in-Chief of this journal.

## Abbreviations

CT: Computed tomography; EGD: Esogastro-duodenoscopy; EST: Endoscopic sclerotherapy; EVL: Band ligation of esophageal varices; MRI: Magnetic resonance imaging.

## Competing interests

The authors declare that they have no competing interests.

## Authors’ contributions

ML was involved in data acquisition, analysis and interpretation, and in drafting the manuscript. DAI was involved in data analysis and in the critical revision of the manuscript. Both authors read and approved the final manuscript.
